# A pilot study to evaluate the serum Alpha-1 acid glycoprotein response in cats suffering from feline chronic gingivostomatitis

**DOI:** 10.1186/s12917-020-02590-2

**Published:** 2020-10-15

**Authors:** Lisa A. Mestrinho, Rita Rosa, Patrícia Ramalho, Vasco Branco, Leonor Iglésias, Hugo Pissarra, Ana Duarte, Maria Niza

**Affiliations:** 1 CIISA - Centro de Investigação Interdisciplinar em Sanidade Animal, Faculdade de Medicina Veterinária, Universidade de Lisboa, Avenida da Universidade Técnica, 1300-477 Lisbon, Portugal; 2 Faculdade de Medicina Veterinária, Universidade de Lisboa, Avenida da Universidade Técnica, 1300-477 Lisbon, Portugal; 3 Research Institute for Medicines (iMed.ULisboa), Faculty of Pharmacy, Universidade de Lisboa, Av. Prof. Gama Pinto, 1649-003 Lisbon, Portugal

## Abstract

**Background:**

Feline chronic gingivostomatitis (FCGS) is a multifactorial immune-mediated disease that can lead to chronic pain, anorexia, and weight loss and has substantial health and welfare effects. Currently, the recommended treatment includes dental extractions to decrease the inflammatory stimulation associated with dental plaque. However, complete remission is observed in less than half of the cases, and the majority need comprehensive medical management. This study aimed to evaluate the serum levels of the acute phase protein alpha-1 acid glycoprotein (AGP) in cats with FCGS and to examine whether dental extractions contribute to a significant decrease in the systemic inflammatory response at two postoperative time points.

**Results:**

AGP serum concentrations in the cats with FCGS were significantly higher at all time points than that in the control groups and were significantly correlated with the global caudal stomatitis score at day 0 but not at day 30 or 60. A significant improvement of some clinical scores, such as perceived comfort and global caudal stomatitis, was observed 60 days after the dental extraction. However, the levels of AGP did not significantly change over time.

**Conclusions:**

Cats with FCGS were more likely to have a systemic inflammatory response compared with age- and dental disease-matched controls. Dental extractions, in most cases, did not contribute to a significant decrease of AGP both at 30 and 60 days. Therefore, this study reinforces the need to pursue comprehensive medical management after dental extractions to attenuate the systemic inflammatory response as a result of this disease.

## Background

Feline chronic gingivostomatitis (FCGS) is a severe multifactorial inflammatory disease that has a limited response to the current treatments [1]. The role of feline calicivirus (FCV) is yet to be clarified in the pathogenesis of this disease, although several authors theorize that FCV can act as an immunologic trigger that disrupts an aberrant immunological response [1, 2].

Currently, the recommended treatment includes dental extractions to decrease the inflammatory stimulation associated with dental plaque [3]. Studies report 37% of cured animals with either partial or total extractions and significant improvement or cure in 50 to 67% of cases [2, 4]. Although dental extractions are associated with some symptom improvement, no therapy allows for complete remission [1], and more than 68% of treated animals require extended medical treatment [4]. One study showed that 7% of the affected cats that do not respond to treatment presented higher caudal and alveolar stomatitis scores, not related to FCV load [2].

FCGS has a significant effect on systemic health, leading to persistent anorexia and weight loss [3]. Polyclonal gammaglobulinemia is persistently observed in these animals [3, 4], suggesting that there is a systemic effect that goes beyond local inflammation. Indeed, a previous report in cats with advanced periodontal disease concluded that the significant local inflammation could influence systemic responses and organ function in distant sites [5]. However, the systemic inflammatory response in cats affected with this disease has never been evaluated; in particular, it is not known if dental extractions can effectively decrease such a state. Measurement of acute-phase proteins (APPs) may be a valuable tool to evaluate the contribution of FCGS to systemic disease, providing a potential mechanistic link between FCGS and its effects, as previous reports in dogs with periodontal disease have shown [6, 7].

APPs help to restore homeostasis and eliminate the cause of a disturbance, infection, trauma, or even a tumor. Alpha-1 acid glycoprotein (AGP), along with serum amyloid A, is one of the positive APPs used to monitor various diseases in cats, including infectious peritonitis and lymphoma. It is stimulated by the increase of pro-inflammatory cytokines such as interleukin (IL)-6, IL-1 beta, tumor necrosis factor (TNF)-alpha, and interferon (IFN)-gamma [8–11]. AGP belongs to the lipocalin family, shows immunoregulatory and antithrombotic properties, transports small hydrophobic molecules, and binds to plasma proteins and mediators, acting as a chaperone in the regulation of innate defense [12, 13].

We hypothesize that AGP is increased in cats with FCGS compared to controls and that dental extractions contribute to a significant decrease in the systemic inflammatory burden. The specific objectives of this study were as follows: (1) to compare the AGP levels between cats with FGCS and matched controls; (2) to evaluate and compare the AGP levels at 30 and 60 days after the dental extractions.

## Results

All cats were domestic shorthair. Control group 1 was composed of three males and seven females, with a mean age of 7.6 months (range: 6 months to 1 year) and a mean weight of 3.2 kg. Control group 2 was composed of three males and five females, with a mean age of 6.8 years (range: 1–15 years) and a mean weight of 4.31 kg. The diseased group was composed of five males and five females, with a mean age of 7.3 years (range: 2–13 years) and a mean weight of 3.90 kg. Control group 1 was significantly younger than control group 2 and the diseased group. The mean age was not statistically different between control group 2 and the diseased group.

In both control groups, all cats tested negative for FCV. In control group 1, no dental anomalies were apparent. In control group 2, periodontal disease and tooth resorption were frequently observed. The average number of tooth resorptions diagnosed per cat was 4.2, with a range of 0 to 10. Periodontal disease stage 1, 2, 3, and 4 was diagnosed in five, three, one, and one cat, respectively. A dental fracture was diagnosed in one cat.

**Fig. 1:**
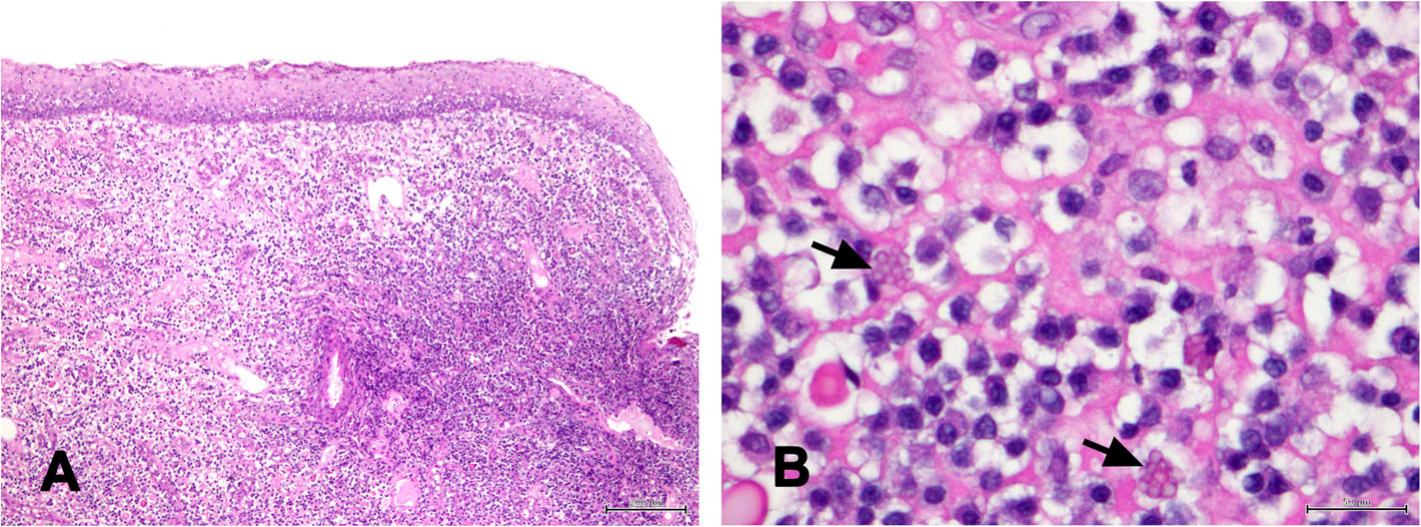
**a** Low (100x) and (**b**) high (400X) hematoxylin and eosin magnification images of gingival sections from cats with FCGS showing grade 3 inflammation severity, with intense infiltration of the gingival mucosal chorion by plasma cells, lymphocytes and neutrophils (**a** and **b**). Mott cells (black arrow) could also be found

In the diseased group, the reported chronicity of the clinical signs was more than 6 months. In eight cats, tooth resorptions were diagnosed radiographically. All cats were positive for FCV, and all biopsies were classified as having grade 3 inflammation severity, which is characterized by intense infiltration of the gingival mucosal chorion by plasma cells and lymphocytes. Infiltration by neutrophils was observed in half of the cases. Mott cells (B lymphocytes with immunoglobulins) were frequently detected (Fig. 1).

Regarding the treatment received by the diseased group, partial extractions (all premolars and molars) were performed in seven cats, and total extractions were performed in three. At day 60, response to treatment was as follows: EOT0 - two cases, EOT1 – three cases, EOT2 – three cases, and EOT3 – two cases. In half of the cases, the treatment was considered successful (EOT 2 and 3). The type of treatment (full mouth vs. partial extractions) was not related to a successful or unsuccessful outcome (*p* > 0.99).

**Fig. 2:**
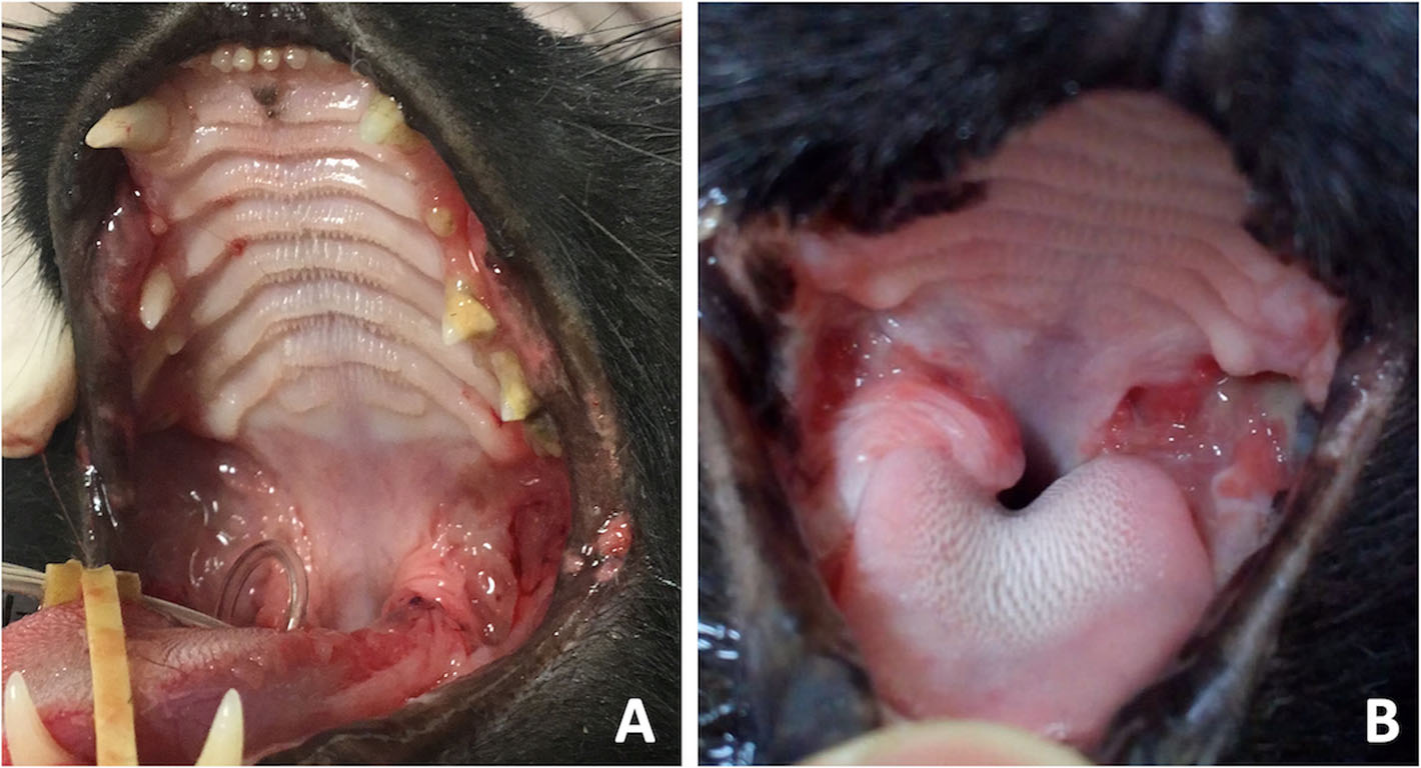
Photograph of one cat from the diseased group at day 0 (**a**) and day 60 (**b**) after dental extractions

The global caudal stomatitis intensity score (GCSIS) significantly decreased at the end of 60 days, although the isolated caudal and alveolar stomatitis score improvements at the same time point were not statistically significant (*p* = 0.052 and *p* = 0.092, respectively; Fig. 2). Grooming behavior and perceived comfort scores significantly improved 60 days after partial or full mouth extraction (*p* = 0.027 and *p* = 0.019, respectively). Appetite and activity level scores did not improve significantly after 60 days (*p* = 0.14 and *p* = 0.39, respectively). Weight also did not show any significant improvement.

**Fig. 3:**
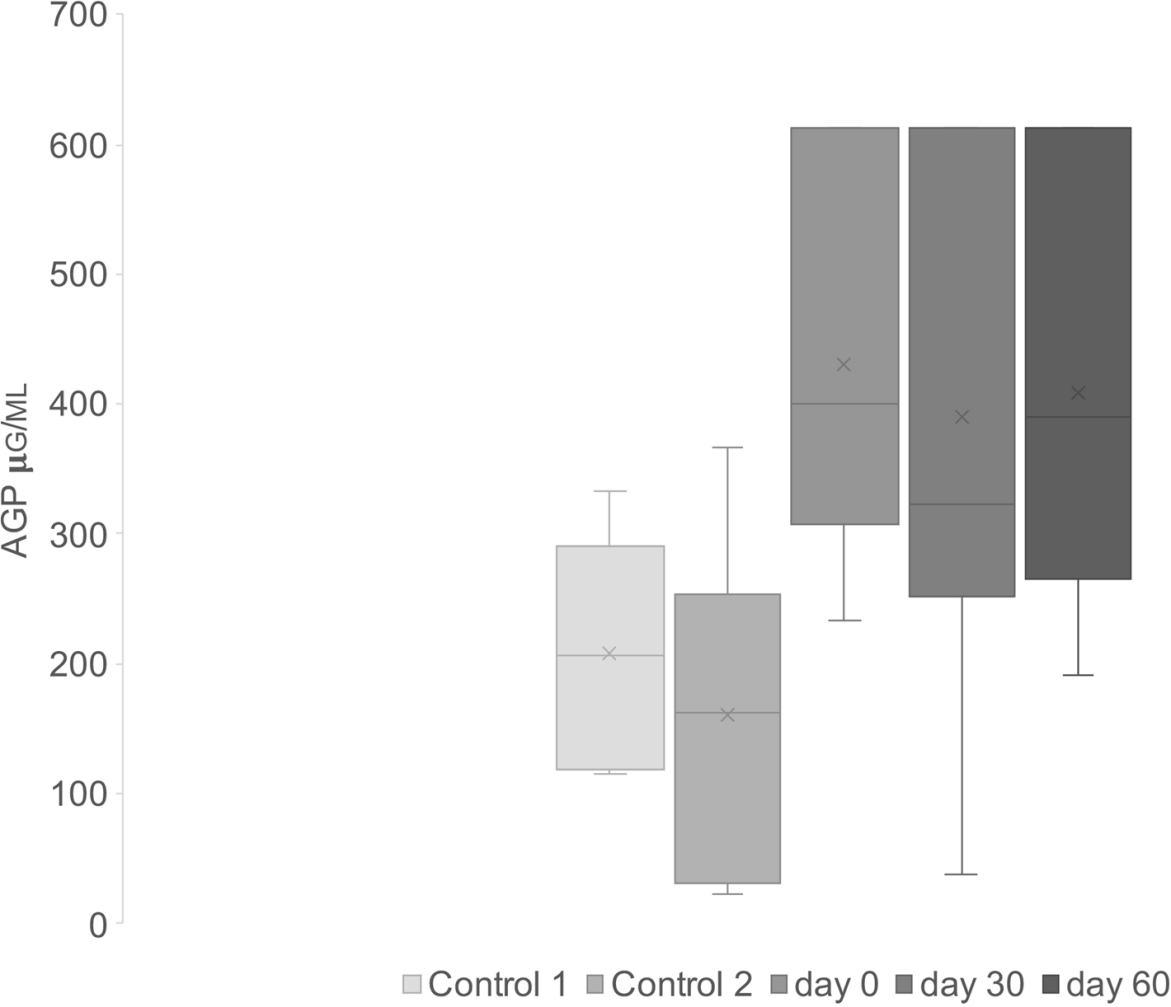
AGP levels in control and disease groups (d0, d30 and d60)

In the diseased group, six AGP measurements exceeded the upper limit of the quantification method (612 μg/ml). These maximum values were assumed as definitive in the statistical analyses since samples were diluted twice and further dilution was not done. AGP serum concentrations in the FCGS cats were significantly higher than that of controls (430.6 μg/ml, 95% confidence interval [CI] [324.94, 536.26]; and 181.67 μg/ml, 95% CI [130.43, 232.91], respectively; *p* = 0.000018). The AGP serum levels for the first and second control group were 207.25 (95% CI [147.57, 266.93]) and 161.2 (95% CI [89.90, 232.50]), respectively. No statistical differences were found between both control groups (*p* = 0.36). Significant differences were observed between the diseased group and both control groups (*p* = 0.000088; Fig. 3).

At both time points (day 30 and day 60), AGP was significantly higher than that in the first control group (*p* = 0.033 and *p* = 0.0055, respectively) and second control group (*p* = 0.0069 and *p* = 0.00088, respectively; Fig. 3).

**Fig. 4:**
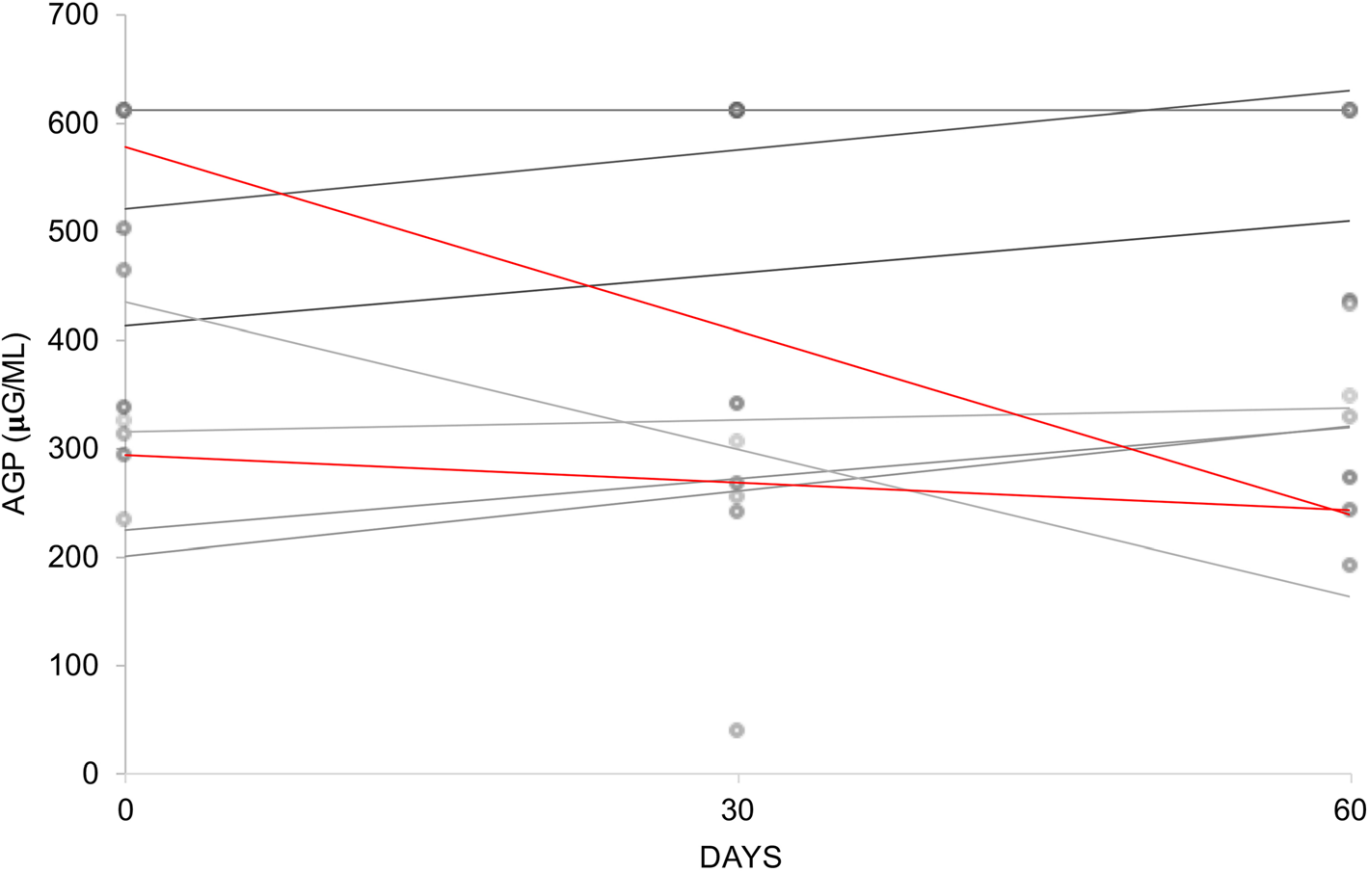
AGP dispersion plots for each case. Red lines corresponded to the 2 cases with complete cure after dental extractions (EOT3)

At day 30, a decrease of AGP in four cases was observed, three of which had a favorable treatment response. In three cases, AGP increased at day 30, while two did not respond to treatment. At day 60, the AGP levels decreased in three cases compared with that at day 0, which showed a favorable response to the dental extraction (Fig. 4).

A significant positive association between AGP serum concentration and GCSIS was observed at day 0 (r(10) = 0.68, *p* = 0.031) and not at the remaining time points. No significant association was found between a decrease of AGP and successful vs. unsuccessful outcomes (*p* = 0.17).

## Discussion

We aimed to test the hypothesis that FCGS is associated with a systemic inflammatory state and evaluate whether dental extractions would significantly reverse this burden.

The study design was prospective using two control groups without changes in routine pre-operative blood analysis. The first control group, without FCGS, was significantly younger than the diseased group. This choice resulted from the need to have a control group without dental disease since dental disease incidence increases with age. Therefore, an older control group was necessary to age match with the diseased group. In this second group, there was an absence of other systemic comorbidities that could induce systemic inflammation, which was confirmed with the clinical history and blood analysis. To assess dental disease as a confounding factor, the second control group included animals submitted to routine dental treatment. These animals had no history of dental pain, but mild to moderated dental anomalies were observed in the routine consultation, and the owners were advised to pursue dental treatment. Two control groups are, from the authors’ point of view, considered an advantage, since they allow for a better appreciation of the systemic effect of FCGS compared with other common dental diseases. At the same time, it was possible to match the effect of age and dental disease (periodontal disease and tooth resorption), which are found not only in cats affected with FCGS but also in older animals [14, 15]. In both control groups, the AGP values obtained were within a similar reference range as reported by others [11, 16], and no significant differences were observed between them. This result can be an effect of the low number of animals in each group or because only severe dental diseases can lead to a systemic effect, quantifiable in terms of acute-phase reactants. A previous study demonstrated that severe periodontitis was associated with systemic changes in total globulins, albumin, and cholesterol in cats [5]. Moreover, no positive APPs were determined, but globulin, albumin, and cholesterol were found to be directly related. To the best of our knowledge, the systemic levels of AGP in tooth resorption or periodontal disease have not been previously reported. However, this group of cats did not present severe dental disease. Future studies should evaluate the levels of AGP in different dental diseases and stages.

All animals were positive for FCV and presented grade 3 inflammation severity. This was an expected result since part of this inflammatory condition is driven by FCV [16]. This study cannot, however, due to the absence of a positive control (diseased FCV negative group), completely exclude the possibility that the AGP concentration values in the diseased group were a result of the combination of FCV infection and inflammation. On one hand, the pathophysiology of FCGS is still unclear. The viral load does not seem to be associated with the severity of lesions [2], but, its prevalence is high, varying from 40 to 90% [17–21]. On the other hand, experimental attempts to infect cats with FCV failed to reproduce FCGS [22, 23]. Finally, FCV prevalence in healthy cats is very low, ranging from 0 to 8% [18, 24]. These facts explain the lack of FCV positive results in the control group, which is one of the intrinsic limitations of this study. Indeed, it would be ideal to evaluate two diseased and two control groups with and without FCV infection to allow for the comparison of the APP levels and appreciate the effect of FCV alone in acute phase reaction. That said, due to the intimate association of the virus in the etiopathogenesis of FCGS, it is challenging to find FCV carriers without signs of oral disease and even more difficult to form a diseased FCV negative group.

Our results confirmed that the serum AGP levels are significantly increased in cats with FCGS, suggesting that this chronic oral inflammation is related to, or may induce, a systemic inflammatory state. Elevated concentrations of APPs have also been described in several other acute and chronic inflammatory diseases [8, 9, 25, 26]. APPs are produced by the liver in response to cytokines such as IL-1, IL-6, and TNF-α, which are mostly elevated in patients with FCGS [27]. The systemic leakage of these mediators leads to a persistent acute phase response that results in metabolic changes such as lipid metabolism changes, hypoferremia, increased gluconeogenesis, increased muscle catabolism, activation of complement, and coagulation [28, 29]. Consequently, the effect of a long-standing acute phase response may not be as beneficial as expected, since it can contribute to events such as cachexia and multiple organ lesions secondary to such metabolic changes, with the kidney being a major target organ in this process [30, 31]. Therefore, the systemic inflammatory burden of FCGS should be recognized, since it supports a therapeutic approach that must go beyond the control of clinical signs and minimize the systemic effects of this persistent systemic inflammation.

Although APPs are not disease-specific markers, their sensitivity is high [12]. Regarding oral disease, the intensity of gingival inflammation is proportional to the intensity of the systemic response, as measured by C-reactive protein levels in a study of dogs with periodontal disease [7]. In our study, a positive association between the AGP and stomatitis scores was found, as AGP was significantly associated with GCSIS at day 0. GCSIS considers the surface area of inflammation, and, therefore, it makes sense that a larger amount of affected tissue contributes to greater leakage of cytokines to the system. However, from these results alone, it is not possible to conclude if the intensity and extent of the local inflammation are powerful enough to induce such a severe increase of APPs or if the increase is due to an autoimmune/auto-inflammatory phenotype.

In our study, the AGP serum levels did not significantly change at day 30 and day 60 after the dental extractions, except in the two cases that achieved a cure. In the remaining cases, an increase of AGP was observed from day 0 to day 60 (four cases), was persistently above the upper limit of quantification (three cases), or remained unchanged (one case). In cases with poor response to dental extractions, the levels of AGP were above the maximum level of absorbance.

The observations in this study confirm that the systemic inflammatory response to dental extraction alone, after 30 and 60 days, is limited. In one study on healthy cats with experimentally induced inflammation, the AGP levels started to decrease 3 days after the injury, achieving comparable values with those at the pre-operative state 11 days after surgery [8]. If we consider FCGS, it would be expected that the effect of surgery could potentiate the previously established inflammation and lead to an extension of the AGP elevated levels for a longer period. This potentiated effect would be controlled by the medical management (which included anti-inflammatory medication) and explain why the systemic inflammatory response decreased in four cases.

Therefore, the post-operative medical treatment prescribed after dental extraction could be related to the AGP decrease at day 30 as observed in four cases. However, at the same time point, an increase was observed in three cases. In theory, if the inflammatory trigger is eliminated (dental extractions), and based on the time for the AGP levels to decrease to normal values in healthy cats [8], the response to dental extractions would not take longer than 30 days. If we look at the five cases that responded poorly to the dental extractions (Additional file 1: Supplementary table), the AGP values increased from day 0 to day 60 in three cases or were above the maximum level of absorbance. In these cases, the effect of the extractions to remove the inflammatory trigger did not influence the disease itself and just contributed temporarily to an increase of the established inflammation.

The benefit of dental extractions has been demonstrated in previous clinical studies [1, 2, 4]. However, complete remission only occurs in approximately a third of such cases, and more than 68% of treated animals need comprehensive medical management [2, 4]. This exploratory study shows that the systemic inflammatory state is not significantly reversed by the dental extractions alone, at least 60 days after the procedure. In addition, in some cases, the treatment response is not favorable, meaning that high levels of acute-phase reactants will persist for long periods of time.

Therefore, monitoring the animals’ acute-phase profile may serve as a clinical indicator of the magnitude and direction of the inflammatory disease process. This is of the utmost importance since persistent chronic inflammation, particularly severe dental disease, can lead to an increased risk of distant organ injury, such as chronic renal failure [32, 33].

Further studies are needed to establish a mechanistic link between FCGS, systemic inflammation, and its consequences.

## Conclusion

In conclusion, the systemic inflammatory response, evaluated through AGP measurements, is elevated in cats affected with FCGS compared with matched controls. Moreover, dental extractions, in most cases, did not contribute a significant decrease of AGP at 60 days. Therefore, the results from this work suggest that dental extractions cannot be a “stand alone” treatment for FCGS due to the persistent inflammatory burden associated with the disease and reinforces the need to medically control this chronic inflammatory state in order to attenuate its consequences in distant organs.

## Methods

### Study design and patient selection

The study was approved by the Ethics and Welfare committee of the Faculty of Veterinary Medicine, Lisbon University (Reference number 012/2018). All cats included in the study were client-owned cats, admitted for consultation and subsequent surgical procedure, which were included in the study after the informed consent was signed by the owner, from August 2018 to August 2019.

A total of 28 cats were included in the study: 10 cases and 18 controls.

The control group, cats without FCGS, was divided in 2. The first control group, gender matched, included cats, younger than 2 years of age, presented for elective surgery, showing no signs of any dental or systemic disease. The second control group, age and gender matched, included those anesthetized for routine dental treatment, with other dental diseases but not showing signs of systemic disease (confirmed by pre-operative blood work).

In all cats the pre-operative blood work (hemogram and basic biochemistry - total proteins, albumin, urea and creatinine) were within normal range and all tested negative for retroviral disease - feline immunodeficiency virus and feline leukemia virus.

The diseased group included cats with FCGS without apparent comorbidities following physical examination and blood testing, except those directly related with the disease, such as hyperglobulinemia.

Exclusion criteria for both groups were the presence of co-morbidities with influence on AGP - neoplasia, renal or hepatic disease, retroviral disease, among others. Animals recently vaccinated (less than 15 days between vaccination before blood testing), recently treated with immunosuppressive drugs and those that developed systemic alterations during the study were also excluded.

All cats underwent a comprehensive oral and dental examination at the time of blood collection for immunoassays, which was performed during the pre-operative blood sampling. After sedation, intra-oral radiographs were performed in both groups as well as an oral swab was collected for FCV analysis. FCV nucleic acid was extracted and amplified by reverse-transcription polymerase chain reaction as previously described [34].

### Clinical evaluation

Periodontal disease was staged as advised by the American Veterinary Dental College Nomenclature Committee (2020) into grade 1 to 4 (http://avdc.org/nomenclature.html, Accessed 26th March 2020). Oral inflammatory lesions were graded by the same operator, using a previously reported scoring system [2]. Caudal and alveolar stomatitis intensity score were evaluated using a 5 degree system: Grade 0 corresponds to no lesion; grade 1 to mild inflammation, non-ulcerative, non-proliferative, not spontaneously bleeding, not bleeding when applied slight pressure; grade 2 moderate inflammation, non-ulcerative, slightly proliferative or not, no spontaneously bleeding nor when applied slight pressure; grade 3 moderate, ulcerative or ulcero-proliferative inflammation, without spontaneously bleeding, but bleeding when a slight pressure is applied, and grade 4 severe, ulcerative or ulcero-proliferative inflammation with spontaneous bleeding. Surface area caudal stomatitis was scored in 5 degrees: 0 absence of lesions, 25 if < 25% of total surface area, 50 if 25–50% of surface area, 75 if 50–75% surface area and 100 for > 75% surface area. Finally, the global caudal stomatitis intensity score (GCSIS) was calculated according with the formula (global caudal intensity score X surface score)/100.

Clinical response to treatment in terms owner evaluation was evaluated at all time points in a scale from 0 to 3 for each of the parameters: appetite, activity level, grooming behavior and perceived comfort, as reported previously [35].

### Histopathology

Biopsies were performed in all animals from the diseased group, from most representative areas, caudally located. Lesions were scored 0 to 3 according with previous reported [36] Grade 0: normal, 1: minimal to mild, 2: moderate, 3: severe inflammation.

### Surgical treatment

The surgical treatment consisted in removal of the dental calculus and polishing of teeth, followed by extractions of all those presenting more than 50% of periodontal attachment loss, pulpal lesions and tooth resorption. In the diseased group partial extraction defined when all premolars and molars were extracted.

Post operatory prescriptions included 2 weeks antibiotic therapy, clindamycin (Clindaseptin, Chanelle Pharmaceuticals, Ireland), 8 days analgesia, buprenorphine (Bupaq, Richter Pharma, Austria), 5 days non-steroidal anti-inflammatory, meloxicam (Metacam, Boehringer Ingelheim, Germany) and a plaque control gel (Clunia Clinical Zn-A-gel, VetNova, Spain) for 15 days.

### AGP analysis

Peripheral blood was harvested aseptically to a dry tube from the cephalic vein. Serum was obtained following centrifugation of blood at 7000 rpm for 5 min and stored at − 20 °C until further processing. In the control group, only one blood sample was collected at the time of enrolment in the study (day 0). In the diseased animals, three samples were collected at different time points: immediately after sedation (day 0), at day 30 and day 60 after the surgical treatment. Measurement of AGP was performed in all samples using a commercially available ELISA kit (AGP-8, Life Diagnostics, USA). This assay uses affinity purified cat AGP antibodies for solid phase immobilization (microtiter wells) and horseradish peroxidase conjugated antibodies for detection. In the presence of AGP molecule bound to the immobile phase and by reaction with the detection antibodies, the well develops the blue color. Color development is halted by addition of a stop solution, changing the color to yellow. Thereafter, the absorbance at 450 nm is measured in the spectrophotometer (Anthos Zenyth 3100, Labtec Instruments). The concentration of the protein is proportional to absorbance being derived from a standard curve (Additional file 2: Supplementary graphic).

### Treatment evaluation

Effectiveness of the treatment (EOT) was assessed according to previously reported [4]. EOT of 0 when there was no favorable evolution or there was clinical aggravation after total or partial extraction, EOT of 1 when was observed slight improvement after partial or total extractions, with some clinical signs remaining, EOT of 2 when there was a significant improvement, after partial or total extractions and no apparent clinical signs, EOT of 3 which corresponded to clinical cure, when there was a complete resolution after partial or total extraction. The classification was also grouped in failure versus success corresponding to the grouping of the classifications 0 and 1 versus 2 and 3, respectively.

### Statistical methods

Descriptive statistics calculations were performed using a commercial software calculator package **(**Microsoft Excel version 16.28 for MAC, Microsoft Office, 2018, USA). For inferential statistics calculations an open source statistical software package (R: a language and environment for statistical computing, version 3.5.1 (2018-07-05) R Foundation for Statistical Computing, Austria) was used. For continuous variables (age, weight and AGP) differences between groups were assessed with the T-test or Mann-Whitney when normality was not assured. For the comparison of AGP between timepoints a one-way ANOVA test for repeated measures was used or Mann-Whitney when normality was not assured. To evaluate correlation between AGP and GCSIS, the Spearman’s test was used.

For the analysis of categorical variables (clinical scores and treatment response) the nonparametric Wilcoxon rank and Fisher’s exact test were used. Finally, Phi coefficient was calculated for dichotomous variables (success/unsuccess, increase/decrease). The differences between groups were considered statistically significant when the *P* value (level of significance) was less than 0.05, for a 95% confidence interval.

## Supplementary information

**Additional file 1: Table S1.** AGP serum values of the diseased group at day 0, 30 and 60, according with outcome (EOT). Cases identified with ↓ corresponded to a progressive decrease of the proteins through the 3 timepoints.

**Additional file 2.** Supplementary graphic – Calibration line prepared with standard values for the spectrophotometer.

## Data Availability

The datasets used and/or analyzed during the current study are available from the corresponding author on reasonable request.

## Abbreviations

AGP: Alpha-1 Acid Glycoprotein
APPs: Acute Phase Proteins
CI: Confidence Interval
EOT: Effectiveness Of the Treatment
FCGS: Feline chronic gingivostomatitis
FCV: Feline Calicivirus
GCSIS: Global Caudal Stomatitis Intensity Score
IL: Interleukin
TNF: Tumor Necrosis Factor
