# Supplementary material for: A pilot study to evaluate the serum Alpha-1 acid glycoprotein response in cats suffering from feline chronic gingivostomatitis
Source: BMC Vet Res. 2020 Oct 15;16:390. doi: 10.1186/s12917-020-02590-2 (PMC7558631; doi:10.1186/s12917-020-02590-2)
Supplement: Supplementary file 1 — Additional file 1: Table S1. AGP serum values of the diseased group at day 0, 30 and 60, according with outcome (EOT). Cases identified with ↓ corresponded to a progressive decrease of the proteins through the 3 timepoints. [file 12917_2020_2590_MOESM1_ESM.docx]

| **Cat** | **AGP (µg/ml)** | | |  | **EOT** | **Treatment response** |
| --- | --- | --- | --- | --- | --- | --- |
|  | **0** | **30** | **60** |  |  |  |
| #1 | 612 | 612 | 612 |  | 0 | Failure |
| #9 | 326 | 305 | 348 |  | 0 | Failure |
| #5 | 233 | 255 | 328 |  | 1 | Failure |
| #6 | 612 | 612 | 612 |  | 1 | Failure |
| #8 | 503 | 612 | 612 |  | 1 | Failure |
| #3 | 338 | 612 | 435 |  | 2 | Success |
| #10 | 312 | 38 | 432 |  | 2 | Success |
| #7 | 464 | 241 | 192 | ↓ | 2 | Success |
| #2 | 294 | 268 | 243 | ↓ | 3 | Success |
| #4 | 612 | 341 | 272 | ↓ | 3 | Success |
