# Supplementary figures and images for: A pilot study to evaluate the serum Alpha-1 acid glycoprotein response in cats suffering from feline chronic gingivostomatitis
Source: BMC Vet Res. 2020 Oct 15;16:390. doi: 10.1186/s12917-020-02590-2 (PMC7558631; doi:10.1186/s12917-020-02590-2)

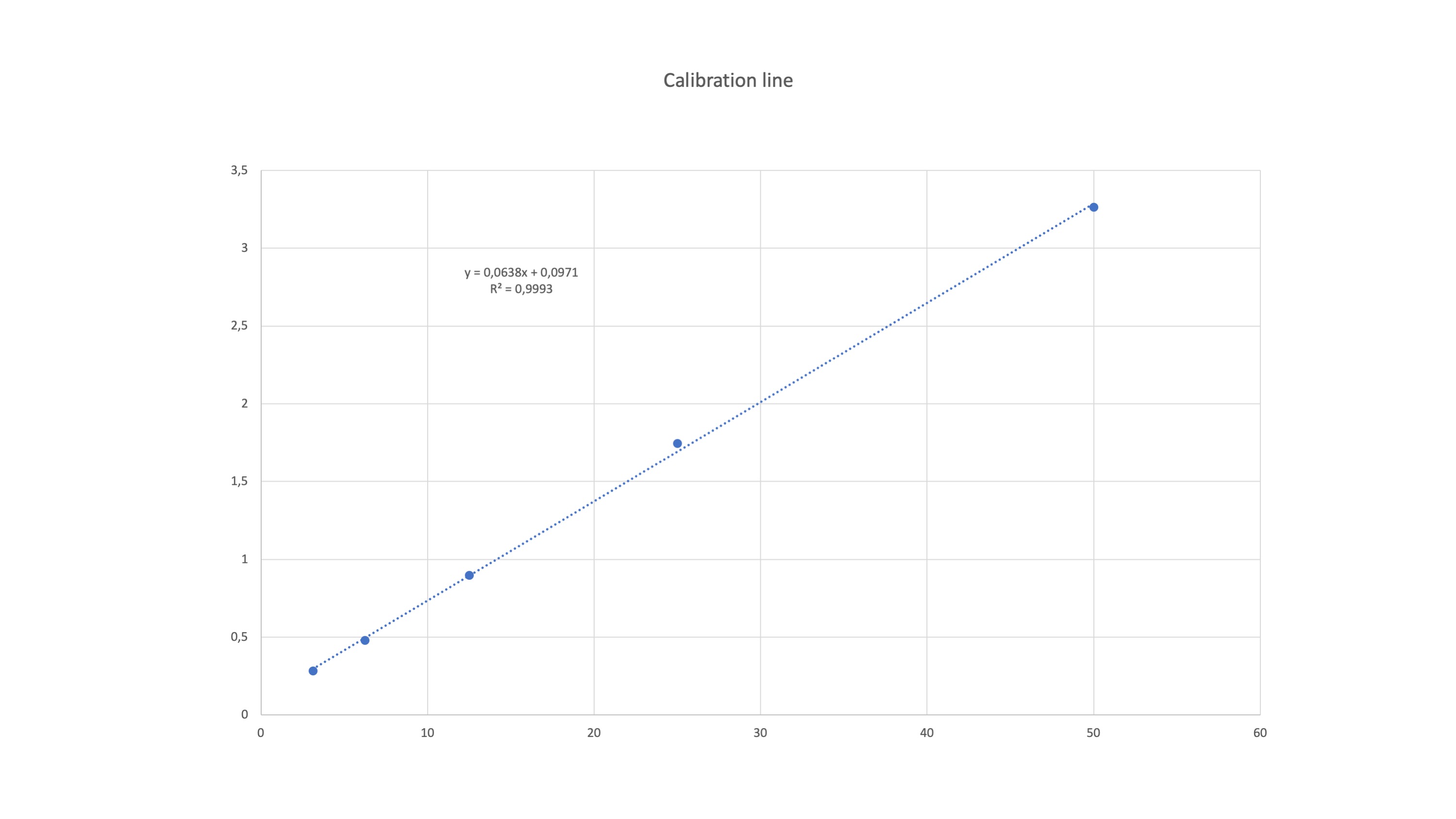

Supplement: Supplementary file 2 — Additional file 2. Supplementary graphic – Calibration line prepared with standard values for the spectrophotometer. [file 12917_2020_2590_MOESM2_ESM.jpg]
